# Postpartum Seizure and Subarachnoid Haemorrhage Secondary to Moyamoya Disease

**DOI:** 10.1155/2019/6132835

**Published:** 2019-12-24

**Authors:** L. Chitra Varanasi, James Brown, Neil Athayde

**Affiliations:** Department of Women's and Newborn Health, Westmead Hospital, Cnr Hawkesbury and Darcy Rd, Westmead, NSW 2145, Australia

## Abstract

Postpartum seizures secondary to subarachnoid haemorrhage (SAH) are rare. The incidence of pregnancy-related SAH is increasing and is highest during the delivery and postpartum periods. While there have been cases in the literature of SAH occurring postpartum, very few are associated with Moyamoya disease. We present a rare case of a young woman diagnosed with Moyamoya disease following immediate postpartum seizures secondary to a SAH. She was medically managed and discharged without any neurological deficits. This case highlights how seizures and SAH may develop in the immediate postpartum period in an otherwise healthy young woman.

## 1. Introduction/Background

Moyamoya disease is a chronic, progressive cerebrovascular disease of the internal carotid arteries and the main branches causing narrowing or blockage that leads ischaemic and haemorrhagic strokes [1]. It is a rare condition affecting children and adults, with a particular predominance in the Japanese population [2].

It has been described in pregnant women [3]. However, we describe a unique case of an immediate postpartum complication. A young nulliparous Indian woman, with no previous medical conditions and an unremarkable antenatal history, suffered a generalised tonic clonic seizure immediately after giving birth. CT (computed tomography) brain and angiography revealed a subarachnoid haemorrhage (SAH) with stenosed intracerebral arteries consistent with Moyamoya disease.

## 2. Case Presentation

The patient is a 28-year-old, gravida 1 para 0, woman who had an unremarkable antenatal course.

She presented to the birth unit at 39 weeks and four days of gestation in spontaneous labour. She had a normal intrapartum blood pressure of 110/80 and did not have an epidural. She had a normal vaginal delivery after a short second stage of 34 minutes and a total duration of labour of five hours and fifteen minutes. The birth was complicated by a postpartum haemorrhage of 1050 mL due to her episiotomy wound.

Immediately after birth, when ten units of intramuscular oxytocin was given and prior to delivery of the placenta, a generalised tonic clonic seizure occurred. The self-limited seizure lasted approximately two minutes followed by a ten minute post ictal phase. Her blood pressure postseizure remained normotensive at 131/84. The patient denied any preceding neurological symptoms, but subsequently complained of a frontal headache. She had an unremarkable neurological examination. She was commenced on magnesium sulphate, and a CT scan of the brain was organised to investigate the seizure.

**Figure 1:**
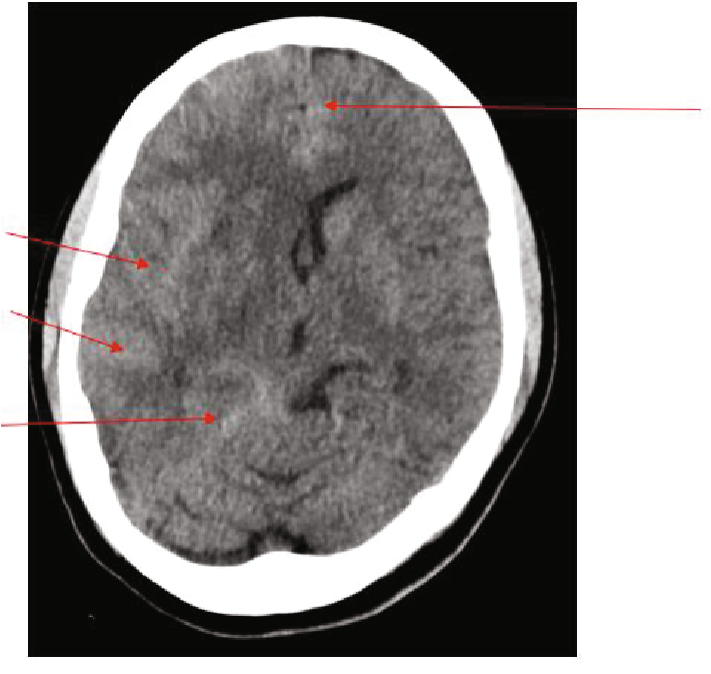
CT brain showing diffused subarachnoid blood with effaced cortical sulcal spaces.

CT brain showed diffuse subarachnoid blood with changes consistent with raised intracranial pressure as demonstrated by the arrows in Figure 1. CT angiogram showed bilateral M1 stenosis suggestive of postpartum angiopathy. Magnesium sulphate was subsequently ceased on neurosurgical review, with a plan to commence IV nimodipine, insert an extraventricular drain (EVD), and perform a digital subtraction angiography (DSA).

A repeat angiogram revealed M1 and ICA (internal carotid artery) stenosis and lenticulostriate collaterals suggestive of Moyamoya disease with distal MCA (middle cerebral artery)/ACA (anterior cerebral artery) irregularity also noted. This demonstrated some improvement following verapamil, suggesting coexistence of reversible cerebral constriction syndrome. She continued IV (intravenous) nimodipine and levetiracetam for treatment of seizures.

She subsequently became febrile. Cerebrospinal fluid aspiration demonstrated numerous polymorphs without bacteria. The EVD was removed, and she was commenced on vancomycin infusion and meropenem for ventriculitis.

Electroencephalogram (EEG) showed evidence of right frontotemporal regional dysfunction on a background of mild diffuse cerebral dysfunction without epileptiform activity. A repeat DSA showed significant improvement in vasospasm with verapamil. She was successfully weaned off IV nimodipine on day 11 and was discharged on day 16 of admission with no neurological sequelae.

## 3. Discussion

Moyamoya disease is prevalent in Japan and East Asia with a higher proportion in females [2]. Children with Moyamoya disease present with motor or speech disturbance and seizures [2]. However, in adults, the typical presentation is headache with altered conscious state due to intracranial bleeding, mostly intraventricular or intracerebral but not subarachnoid [4]. Mortality in the acute stage is up to 16.4%, related to intraventricular or intracerebral bleeding [4].

Only a few cases of Moyamoya disease have been described in pregnancy. A systematic review reported 96 women diagnosed before pregnancy, 23 women during pregnancy, and only 15 postpartum of which seven were diagnosed within three days postpartum [3]. None of these 15 women presented with postpartum seizures as the first presentation of Moyamoya disease [3]. These women presented with ischaemic events (79%) or haemorrhagic events (21%) [3]. Our case uniquely describes a woman who presented minutes after the second stage of labour with a seizure. Significantly, our patient is from India, which to date, has not been published as a common ethnic group susceptible to Moyamoya disease.

Management of Moyamoya disease includes medical treatment for hypertension, vasodilators, anticonvulsants, surgical treatment involving revascularisation surgery, or a combination of these [2, 5]. A recent case series has reported that pregnancy-related stroke secondary to Moyamoya disease is related to gestational age, with the highest risk of intracranial haemorrhage occurring antenatally, particular at or greater than 24 weeks [6]. This is important for clinicians to be aware so increased monitoring for women with known Moyamoya disease can be timed accordingly. There have been no documented cases of SAH due to Moyamoya disease complicated by ventriculitis. Empiric antibiotic therapy, as described above, adequately treated this infection in our patient.

There is no standard for method of delivery in patients with Moyamoya disease [2]. The aim is to maintain haemodynamic stability by controlling blood pressure, managing pain to minimise hyperventilation causing hypocapnia and cerebral vasoconstriction, and careful fluid management [2, 5]. Caesarean section with epidural anaesthetic can be safe, as can an uncomplicated instrumental vaginal delivery [2, 5]. Avoidance of valsalva manoeuvres is recommended [5].

## 4. Conclusion

This is a unique case of a SAH secondary to Moyamoya disease, and our patient was successfully managed and had stabilised with no neurological sequalae prior to discharge. Future pregnancies will require multidisciplinary team involvement to create a thorough antepartum, intrapartum, and postpartum management plan.
